# Challenges in diagnosis and management of giant solitary fibrous tumour of pleura: a case report

**DOI:** 10.1186/s12890-016-0279-0

**Published:** 2016-08-08

**Authors:** Jessica H. Y. Tan, Anne A. L. Hsu

**Affiliations:** Department of Respiratory and Critical Care Medicine, Singapore General Hospital, 20 College Road, Singapore, 169856 Singapore

**Keywords:** Solitary fibrous tumour of pleura, Giant, Haemoptysis, Haemothorax, Case report

## Abstract

**Background:**

Majority of patients with solitary fibrous tumours of the pleura (SFTP) are asymptomatic. Acute presentation with symptoms resulting from mass effect due to rapid expansion of tumour size has not been reported before.

**Case presentation:**

This report chronicles the case of a giant SFTP in a 76-year-old lady who presented with acute onset of haemoptysis, left-sided pleuritic chest pain and hoarseness of voice. Her chest radiograph showed a large left upper hemithorax mass with an ipsilateral effusion. Computed tomography (CT) scan of the thorax confirmed the presence of a pleural-based mass lesion in the left apex measuring 9.7 cm with close apposition to the aortic arch. The mass demonstrated neovascularization and there was also presence of a moderate-sized heterogeneous-appearing left pleural effusion. Thoracocentesis yielded deeply haemoserous pleural fluid with a pleural aspirate hematocrit closely approaching that of peripheral blood hematocrit and alongside a 2 unit decrease in haemoglobin, was indicative of a haemothorax. Repeat CT 10 days from initial presentation showed reduction in size of the left apical mass as well as resolution of the left effusion. This was consistent with the occurrence of an intra-tumoural bleed resulting in rapid increase in the size of the SFTP, causing rupture of superficial blood vessels on the tumour surface (haemothorax) and consequential compression of the lung parenchyma (haemoptysis) and left recurrent laryngeal nerve (hoarseness of voice). The patient eventually underwent an uneventful surgical resection.

**Conclusion:**

A benign SFTP can present acutely with compressive symptoms as a result of spontaneous intra-tumoural bleed causing sudden increase in its size. It is important to allow temporal regression of these acute changes before deciding on surgical resectability.

## Background

Solitary fibrous tumours of the pleura (SFTP) are uncommon tumours of mesenchymal origin. Majority of patients with SFTP are asymptomatic. Haemoptysis and spontaneous haemothorax as initial manifestations are uncommon. A definitive diagnosis requires histopathological/immunohistochemical examination of tumour specimens [1], with the best yield from resected tumours. Complete en bloc resection of the tumour is the mainstay of therapy for SFTP, regardless of it being benign or malignant.

## Case presentation

**Fig. 1:**
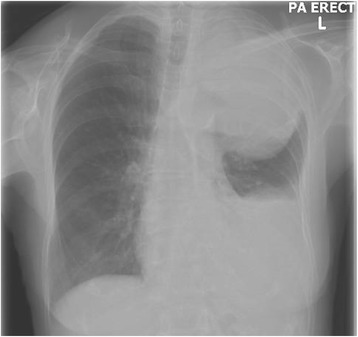
Chest radiograph showed a huge mass in the upper left hemithorax and an ipsilateral moderate-sized pleural effusion

**Fig. 2:**
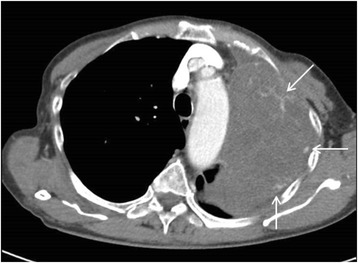
Computed tomography scan of the thorax revealed a large heterogenous mass in the left upper hemithorax with neovascularization (black arrows)

A 76-year-old previously well lady presented to a hospital while on overseas vacation with acute onset of haemoptysis, left-sided pleuritic chest pain and hoarseness of voice that lasted three days. This was associated with weight loss of 7 kg over past two years. She was a lifelong non-smoker, and had no reported history of chest trauma before the onset of her symptoms. She subsequently returned to our centre one week later for further evaluation. Physical examination revealed a thin lady (BMI 13.4 kg/m^2^) who was afebrile with normal blood pressure and pulse rate. Her oxyhaemoglobin saturation on pulse oximetry was normal at 95 % on room air. She had no clubbing or cervical lymphadenopathy. Examination of the lungs revealed stony dullness to percussion in the left lung base with decreased breath sounds throughout the left chest. A radiograph (Fig. 1) performed showed a large, left upper hemithorax mass with an ipsilateral moderate sized left pleural effusion. A computed tomography (CT) scan of the thorax (Fig. 2) confirmed the presence of a large pleural-based mass in the left apex measuring 9.7 × 8.4 × 4.5 cm, with close apposition to the aortic arch, resulting in the collapse of the adjacent lung parenchyma. This mass also demonstrated neovascularization. There was presence of a left pleural effusion containing hyper-dense elements, which suggested the presence of blood or tumour within the pleural space. Laboratory investigation results showed a significant for a drop in haemoglobin of two units from her baseline (12.4 → 10.5 g/dl).

Bedside thoracic ultrasonography revealed a left effusion with heterogeneous appearance. Diagnostic thoracocentesis yielded deeply haemoserous pleural aspirate which was exudative by Light’s criteria [2]. Pleural fluid haematocrit (30.8 %) closely approached that of peripheral blood haematocrit (31.2 %), hence indicative of haemothorax. Pleural fluid cytology was non-diagnostic with rare, atypical cells seen. She further underwent a transthoracic needle Trucut biopsy of the left apical mass, and histopathological features were suggestive of a spindle cell neoplasm (bcl-2 positive, CD34 weakly positive, mesothelial markers CK 5/6, WT1 and calretinin negative) with a mitotic index Ki67 < 5 %.

A subsequent nasoendoscopy also confirmed the presence of left vocal cord palsy, likely caused by the huge mass effect on the intrathoracic component of the left recurrent laryngeal nerve.

The patient’s pre-operative pulmonary function test was normal (FEV_1_ 1.20 L; 105 % predicted and FVC 1.42 L; 81 % predicted) with a moderately reduced diffusion capacity for carbon monoxide (DLCO) corrected for haemoglobin (2.74 mM/min/kPa; 52 % predicted). Her transfer coefficient of the lung (KCO i.e., DLCO/VA; VA = alvelolar volume) was normal, at 80 % predicted.

**Fig. 3:**
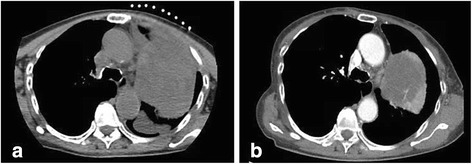
Comparison of the images at the level of the carina during biopsy (**a**) and 10 days later (**b**) showed significant decrease in the size of the left apical mass

**Fig. 4:**
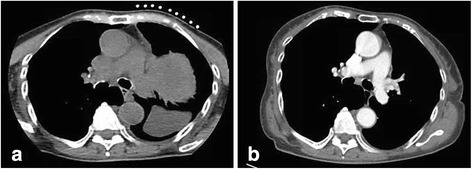
Comparison of the images at a lower level bifurcation of the pulmonary trunk during biopsy (**a**) and 10 days later (**b**) showed complete resolution of the left pleural effusion that gravitated below the mass

At a multidisciplinary chest tumour meeting, thoracic surgeons expressed concerns about the resectability of the mass. This was because of its close apposition to the mediastinum, as well as the possibility of a malignant tumour in the left pleural space which might have invaded the left recurrent laryngeal nerve in an elderly patient with weight loss, and a moderately impaired diffusion capacity which would have deemed her high risk for pneumonectomy if surgically necessary. Based on the opinions of our radiologists and thoracic surgeons and the clincoradiological evidence of intratumoral and pleural haemorrhage of a histological proven SFTP (with no malignant features) in an otherwise well lady, the primary care physicians (the authors of this manuscript) decided to repeat a CT scan about 2 weeks later to reassess the extent of the tumor with regards to surgical resectability. An interval CT scan (10 days apart from the Trucut biopsy; Figs. 3 and 4) showed significant decrease in the size of the mass, and a complete resolution of the pleural effusion. The patient eventually underwent left posterolateral thoracotomy, with excision of the pleural tumour. The post-operative recovery was uneventful, and a CT scan showed re-expansion of the left upper lobe, after resection of the SFTP.

Approximately 1,500 cases of SFTP have been reported in the literature [1, 3]. The majority of patients are asymptomatic at presentation despite a large tumour size. Common symptoms when present include chest pain, cough and dyspnoea [4, 9] (Table 1). Haemoptysis is a rare manifestation, accounting for 2 to 4 % of patients with SFTP in previous reports [4, 5] and appears to be more common in malignant variants [5, 6]. Thus it was unusual for our patient to present with haemoptysis, as her eventual histopathology from the resected tumour did not demonstrate any evidence of malignant transformation. Spontaneous haemothorax associated with SFTP is an even rarer occurrence, with only a handful anecdotal reports in the literature [6–8]. Hoarseness of voice related to SFTP has never been reported.

**Table 1:** Reported clinical manifestations of SFTP [3, 4, 9, 11, 16]

Common	Rare
Symptoms	Symptoms
Chest pain	Fever
Cough	Weight loss
Dyspnoea	Haemoptysis
Signs	Signs
Hypoglycemia	Hypertrophic osteoarthropathy
Pleural effusion	Haemothorax

Our patient’s presentation of sudden onset of haemoptysis, chest pain and hoarseness of voice can be explained by an acute intra-tumoural bleed which caused a rapid increase in size of the SFTP with consequential compressive symptoms. The patient’s haemoptysis could possibly be explained by the huge tumour that caused compression of the adjacent lung parenchyma or airways with distal atelectasis and inflammation [9]. In addition, we postulate that there could be localized pulmonary infarction resulting from the acute mass effect on the pulmonary circulation, which could also lead to the manifestation of hemoptysis. Lung parenchymal inflammation contributed to the patient’s pleurisy, which could also be due to presence of blood in the pleural space. The spontaneous rupture of superficial blood vessels on the surface of the tumour and/or the shear forces from the rapid tumour expansion (due to intra-tumoural bleed) led to haemothorax. The other compressive symptom was her hoarseness of voice, which was possibly a result of acute pressure exerted by the tumour on the left recurrent laryngeal nerve. To our knowledge, this is the first report of acute compressive symptoms caused by intra-tumoural bleed, resulting in rapid expansion in the size of a SFTP. The above postulations on intra-tumoral bleed and haemothorax are supported by an acute drop in her haemoglobin, pleural aspirate haematocrit, and radiological evidence of significant reduction of the left apical mass which demonstrated neovascularisation, as well as the resolution of the left pleural effusion (Figs. 3 and 4) within 10 days. This is likely explained by the natural history of blood resorption within the tumour and pleural space.

Diagnosis of SFTP is challenging due to the lack of specific clinical symptoms and radiographic appearances, as illustrated by the patient in our report who had a rather unique clinical presentation. Differential diagnoses often include bronchogenic carcinoma, malignant mesothelioma and pleural sarcoma. Often, a transthoracic needle core biopsy would be required to differentiate SFTP from other diagnoses. However, the diagnostic yield of needle sampling was variable (ranging from 17 to 45 %) [10–12]. At best, it provides a moderate yield for a definitive pre-operative diagnosis of SFTP. When clinicoradiological features appear to be classically SFTP, most authors advise against a needle biopsy, since it would not alter the eventual management outcome i.e., surgical resection [1, 13].

The mainstay of treatment for SFTP is a complete en bloc resection of the mass [1, 10, 12]. Pedunculated tumours can be safely resected with wedge resection of the adjacent lung. Large sessile tumours can pose a challenge during surgery, and may occasionally require a lobectomy or pneumonectomy in order to achieve complete resection [1]. In a series [11] describing 60 patients with surgical resection done for SFTP, six (10 %) required either lobectomy or pneumonectomy. These features may not be readily apparent in pre-operative radiological imaging studies, and thus there may be no way to predict the extent of lung resection prior to surgery.

There is a paucity of literature on the pre-operative pulmonary assessment of patients requiring surgery for SFTP and large pleural tumours, as existing guidelines [14] only describe pre-operative pulmonary assessment for patients requiring lung resection. Had our patient required a pneumonectomy in the worst-case scenario, her predicted post-operative (PPO) FEV_**1**_ and DLCO would have been 53 and 26 % predicted. The latter value would have rendered her an unacceptable surgical risk [14, 15]. According to the American College of Chest Physician guidelines [14], patients with PPO FEV_**1**_ or DLCO < 60 % predicted have to undergo further physiological assessment with a cardiopulmonary exercise test (CPET) before determining surgical fitness. However, it was noted that her transfer coefficient of the lung (KCO i.e., DLCO/VA; VA = alveolar volume) was normal at 80 % predicted. Her large SFTP had resulted in extrapulmonary reduction in lung inflation, causing a reduced VA. This was largely responsible for the reduction in DLCO [16]. Hence, further pre-operative pulmonary assessment with lung perfusion scan and cardiopulmonary exercise testing for VO_2_ max were not required. The patient subsequently underwent excision of the pleural tumour with minimal lung resection, and had an uneventful post-operative recovery. The risk of recurrence after complete surgical resection can range from <2 % in benign SFTP to 63 % in malignant forms [1].

This case illustrates that KCO can potentially be useful in pre-operative assessment of patients with giant SFTP that results in reduced DLCO, although evidence from larger scale studies would be required to support this hypothesis. It would be very unfortunate had the patient been denied surgery, had she been deemed a high-risk candidate based on her decreased DLCO alone.

## Conclusion

This report showcases a rare manifestation of a benign SFTP with an acute increase in size due to spontaneous intra-tumoural bleeding, which in turn led to haemothorax and compressive symptoms. The simultaneous occurrence of these factors resulted in the earlier presentation of an otherwise slow-growing tumour. It is important to allow temporal regression of these acute changes before deciding on surgical resectability. This case also highlights the potential usefulness of KCO in determining a patient’s pre-operative risk for large pleural tumours that may require lung resection. If pre-operative risk was determined by reduced DLCO alone, patients who need surgery would be denied it.

## Abbreviations

DLCO, diffusion capacity for carbon monoxide; FEV_**1**_, forced expiratory volume in 1 s; FVC, forced vital capacity; KCO, transfer coefficient of the lung; SFTP, solitary fibrous tumour of the pleura; VA, alveolar volume
